# Optimal Threshold of Homeostasis Model Assessment of Insulin Resistance to Identify Metabolic Syndrome in a Chinese Population Aged 45 Years or Younger

**DOI:** 10.3389/fendo.2021.746747

**Published:** 2022-01-05

**Authors:** Szu-Yu Lin, Wen-Cheng Li, Ting-An Yang, Yi-Chuan Chen, Wei Yu, Hsiung-Ying Huang, Xue-Jie Xiong, Jau-Yuan Chen

**Affiliations:** ^1^ Department of Family Medicine, Chang-Gung Memorial Hospital, Linkou Branch, Taoyuan, Taiwan; ^2^ College of Medicine, Chang Gung University, Taoyuan, Taiwan; ^3^ Department of Health Management, Xiamen Chang-Gung Hospital, Xiamen, China; ^4^ Department of Pulmonary and Critical Care Medicine, Xiamen Chang-Gung Hospital, Xiamen, China; ^5^ Department of Oncology, Xiamen Chang-Gung Hospital, Xiamen, China

**Keywords:** metabolic syndromes, HOMA-IR, insulin resistance, diabetes mellitus, cardiovascular diseases

## Abstract

**Background:**

Metabolic syndrome (MetS) is regarded as a major risk factor for diabetes mellitus and cardiovascular disease (CVD). The optimal threshold of the homeostasis model assessment of insulin resistance (HOMA-IR) has been established for predicting MetS in diverse populations and for different ages. This study assessed the serum HOMA-IR level in a healthy Chinese population aged ≤45 years to determine its relationship with metabolic abnormalities.

**Methods:**

Cross-sectional study data were collected from health checkup records of Chinese adults aged ≥18 years between 2013 and 2016 at Xiamen Chang Gung Hospital. Participants completed a standardized questionnaire, which was followed by a health examination and blood sample collection. Exclusion criteria were as follows: history of known CVDs; liver, kidney, or endocrine diseases or recent acute illness; hypertension; hyperlipidemia; and pregnancy or lactation.

**Results:**

The clinical and laboratory characteristics of 5954 men and 4185 women were analyzed. Significant differences were observed in all assessed variables (all P < 0.05). The optimal cutoff point of HOMA-IR for predicting MetS was 1.7 in men and 1.78 in women.

**Conclusions:**

We aimed to determine the optimal cutoff point of HOMA-IR for predicting MetS in a healthy Chinese population aged ≤45 years. The findings of this study would provide an evidence-based threshold for evaluating metabolic syndromes and further implementing primary prevention programs, such as lifestyle changes in the target population.

## Introduction

Metabolic syndrome (MetS) is a cluster of factors that include hypertension, obesity, dyslipidemia, and glucose intolerance. Adult Treatment Panel III (ATP III) has defined the criteria for MetS (1). The presence of MetS or insulin resistance (IR) is related to type 2 diabetes and cardiovascular disease (CVD) (2, 3). The prevalence of MetS in the global population is estimated to be close to 25% (4) and between 7.9% and 35% in the older adult Chinese population (5–7).

IR is a reduced biological response of peripheral tissues to insulin (8). IR plays a major role in a cluster of metabolic features, such as obesity, fat distribution in the abdomen, elevated total cholesterol (T-CHOL), triglyceride (TG), and low high-density lipoprotein (HDL) levels and high blood pressure (BP). This cluster of cardiovascular risk factors is referred to as MetS (3, 9).

The hyperinsulinemic-euglycemic clamp technique is the gold standard for assessing insulin sensitivity (10). Minimal-model analysis of the frequently sampled intravenous glucose tolerance test is another common method (11). However, these methods are expensive and invasive, and they are thus typically reserved for research purposes. To make the assessment of IR more convenient, the homeostasis model assessment of insulin resistance (HOMA-IR) provides an efficient formula for evaluating B-cell function and insulin sensitivity. It is now a widely used and reliable model for assessing IR in epidemiological studies (12, 13).

A review of the literature indicated that the optimal threshold of HOMA-IR has been employed to predict MetS in diverse populations and ages (14–19). However, few studies have focused on the Chinese population, particularly young adults of ≤45 years. This population study assessed serum HOMA-IR levels in healthy Chinese people aged ≤45 years to determine its relationship with metabolic abnormalities, including MetS. Furthermore, we aimed to establish an optimal HOMA-IR value for predicting MetS in this demographic.

## Material and Methods

### Study Design and Participants

We collected cross-sectional data from the health check-up records of Chinese adults aged ≥18 years between 2013 and 2016 at Xiamen Chang Gung Hospital. Participants completed a standardized questionnaire comprising information on history of past illnesses, medications, and physiological conditions, which was followed by a health examination and blood sample collection. Exclusion criteria were as follows: individuals with a history of known CVDs (myocardial infarction, angina, cerebrovascular diseases, heart failure); liver, kidney, endocrine diseases (except MetS) or recent acute illness; hypertension; hyperlipidemia; and pregnancy or lactation. The data were validated by the institutional review board of Xiamen Chang-Gung Hospital, Xiamen, China.

### Anthropometric and Clinical Measurements

Physical examination included measurement of body height (cm), weight (kg), waist circumference (WC) (cm), and BP (mmHg). Body height was measured in a standing position without shoes using a calibrated meter to the nearest 0.5 cm. Weight was measured using a digital scale, according to a standard protocol. WC was measured at the midpoint of the lowest rib and the iliac crest. Body index mass (BMI) was calculated by dividing weight (kg) by height (m) squared.

BP was measured using an automated sphygmomanometer with an appropriate cuff size for arm diameter. Participants were placed in a seated position for at least 15 minutes. Three measurements were obtained at intervals of at least 5 min. The lowest values of the three measurements for systolic blood pressure (SBP) and diastolic blood pressure (DBP) were used in the data analysis. The mean arterial pressure (MAP) was calculated as follows: (2/3) × DBP + (1/3) × SBP.

Metabolically obese but normal weight (MONW) was defined as an individual with a normal BMI (ranging from 18.5 to 23.9 kg/m^2^) or normal WC (<90 cm for men and <80 cm for women), but they have insulin resistance. Overweight was defined as a BMI within the range of 24 to 26.9 kg/m^2^ (24 ≤ BMI ≦ 26.9), and obesity was defined as a BMI greater than 27 kg/m^2^ (6). Venous blood samples were obtained after at least 12 h of overnight fasting. Fasting blood sugar (FBG) levels were measured using an enzymatic reference method. Fasting lipids, including T-CHOL (mmol/L), low-density lipoprotein cholesterol (LDL-C, mmol/L), HDL-C (mmol/L), and TG (mmol/L), were measured using the enzymatic method. Fasting insulin levels were measured using the radioimmunoassay (RIA) method.

### Definition of Metabolic Syndromes and HOMA-IR

Metabolic syndrome was defined following the criteria of the modified National Cholesterol Education Program Adult Treatment Panel III (NCEP ATP III) (20). According to the modified NCEP criteria (20), the presence of any three of the following five factors is required for the diagnosis of metabolic syndrome: TG level ≥ 150 mg/dL or drug treatment for elevated triglyceride levels; high-density lipoprotein cholesterol (HDL-C) level < 40 mg/dL (men) or < 50 mg/dL (women) or drug treatment for reduced HDL-C levels; BP ≥ 130/85 mmHg or antihypertensive drug treatment in patients with a history of hypertension; fasting glucose level ≥ 100 mg/dL or drug treatment for elevated glucose levels; and waist circumference greater than the suggested cutoff point. The modified NCEP ATP III criteria suggested that the cut-off points for waist circumference should be ethnic-specific, wherein a cut-off of ≥90 cm in men and ≥80 cm in women should be considered for individuals of Asian origin.

The insulin resistance index was calculated using the HOMA-IR according to the following formula:


$$HOMA-IR=\frac{\text{fasting insulin}\left(\frac{\text{mIU}}{\text{L}}\right)\times FBG\left(\frac{mmoL}{L}\right)}{22.5}$$

### Statistical Analyses

Data for men and women were compared using independent-sample t-tests for continuous data and chi-square tests for categorical data. A receiver operating characteristic (ROC) curve is a graphical plot of the true-positive rate (sensitivity) versus the false-positive rate (100-specificity) for a binary variable across a range of thresholds. In the current study, ROC curves were used to demonstrate the discriminatory ability of an anthropometric index (e.g., WC) over the entire range of possible values in the detection of a cardiometabolic outcome (i.e., diabetes), as quantified by the area under the curve (AUC). The optimal cutoff point for each anthropometric variable in the prediction of a cardiometabolic outcome was established according to Youden’s index. Odds ratios (ORs) were calculated using multiple logistic regression analysis and are presented with 95% confidence intervals. Statistical significance was set at P < 0.05. The data were analyzed using SPSS version 19.0, for Windows (SPSS, Inc., Chicago, IL, USA). Pairwise comparison of ROC curves was conducted using MedCalc for Windows, version 9.38 (MedCalc Software, Mariakerke, Belgium).

## Results

### Basic Characteristics of the Study Subjects According to Gender

Significant differences were observed between men and women in all the assessed variables (all P < 0.05). **Table 1** presents the basic characteristics and prevalence of cardiometabolic risk factors in 5954 men and 4185 women in the study population.

**Table 1:** Basic characteristics of the study subjects.

Variables	Male	Female	p value
	(n=5954)	(n=4185)	
Age (year)	38.66 ± 4.98	37.94 ± 5.33	<0.001
Waist circumference (cm)	86.74 ± 9.03	74.83 ± 7.83	<0.001
Waist-to-height ratio	0.51 ± 0.05	0.47 ± 0.05	<0.001
BMI (kg/m^2^)	24.73 ± 3.42	21.86 ± 2.93	<0.001
SBP (mmHg)	119.97 ± 15.58	106.05 ± 13.12	<0.001
DBP (mmHg)	75.75 ± 11.26	65.12 ± 9.47	<0.001
Mean arterial pressure (mmHg)	90.49 ± 12.25	78.77 ± 10.16	<0.001
Fasting glucose (mmol/L)	5.32 ± 1.32	5.01 ± 0.61	<0.001
Total cholesterol (mmol/L)	5.25 ± 0.96	4.79 ± 0.85	<0.001
Triglycerides (mmol/L)	1.88 ± 1.97	0.91 ± 0.58	<0.001
LDL cholesterol (mmol/L)	3.38 ± 0.86	2.89 ± 0.76	<0.001
HDL cholesterol (mmol/L)	1.19 ± 0.27	1.46 ± 0.32	<0.001
TG/HDL-C	1.78 ± 2.58	0.69 ± 0.63	<0.001
Insulin(mIU/L)	7.41 ± 4.36	6.18 ± 3.09	<0.001
HOMA-IR	1.78 ± 1.25	1.40 ± 0.78	<0.001

Continuous data are shown as mean ± SD and compared using independent-sample t-tests.

BMI, body mass index; SBP, systolic blood pressure; DBP, diastolic blood pressure; MAP, mean arterial pressure; LDL-C, low-density lipoprotein cholesterol; HDL-C, high-density lipoprotein cholesterol; TG, triglycerides; HOMA-IR, homeostasis model assessment of insulin resistance.

### Optimal Cut-Off Points of HOMA-IR in the Prediction of Metabolic Syndrome

The optimal cutoff point of HOMA-IR for predicting MetS was 1.7 in men (sensitivity 0.75%; specificity 0.74%) and 1.78 in women (sensitivity 0.79%; specificity 0.81%). **Table 2** shows the AUC, sensitivity, and specificity according to the optimized cutoff points for cardiometabolic risk factors in the prediction of MetS. The AUC of TG/HDL (AUC 0.86, cutoff point 1.47) in men was a better predictor of cardiometabolic risk factors than that of other variables. In women, both the AUC of TG/HDL (AUC 0.9, cutoff point 0.86) and WC (AUC 0.9, cutoff point 79.75 cm) had higher accuracy for predicting cardiometabolic risk factors than the other variables. A subgroup of overweight and obese individuals (BMI≧24) was analyzed. The AUC of the subgroup is 0.73 in men and 0.78 in women. The subgroup optimal cutoff point of HOMA-IR was 1.99 in men (sensitivity 0.66%; specificity 0.69%) and 2.06 in women (sensitivity 0.76%; specificity 0.73%).

**Table 2:** The areas under ROC curve (AUC), sensitivity and specificity by the optimized cut-off points for cardiometabolic risk factors in prediction of metabolic syndrome according to gender and obesity status.

Variables	AUC	(95% CI)	Cut-off point according to Youden’s index	Sensitivity (%)	Specificity (%)
**Male (n=5954)**					
Waist circumstance (cm)	0.85	(0.84-0.86)	89.75	0.83	0.78
Waist-to-height ratio	0.84	(0.83-0.85)	0.53	0.78	0.76
MAP	0.78	(0.77-0.80)	95.83	0.65	0.81
Fasting glucose (mmol/L)	0.75	(0.73-0.76)	5.36	0.59	0.80
TG/HDL ratio	0.86	(0.85-0.87)	1.47	0.86	0.74
HOMA-IR	0.81	(0.79-0.82)	1.70	0.75	0.70
**Subgroup : BMI≧24 (n=3453)**					
HOMA-IR	0.73	(0.71-0.75)	1.99	0.66	0.69
**Female (n=4185)**					
Waist circumstance (cm)	0.90	(0.88-0.91)	79.75	0.94	0.78
Waist-to-height ratio	0.89	(0.87-0.91)	0.49	0.92	0.74
MAP	0.78	(0.75-0.81)	87.17	0.60	0.85
Fasting glucose (mmol/L)	0.81	(0.78-0.84)	5.21	0.74	0.76
TG/HDL ratio	0.90	(0.88-0.92)	0.86	0.84	0.81
HOMA-IR	0.88	(0.86-0.90)	1.78	0.79	0.81
**Subgroup : BMI≧24 (n=860)**					
HOMA-IR	0.78	0.74-0.82	2.06	0.76	0.73

BMI, body mass index; SBP, systolic blood pressure; DBP, diastolic blood pressure; MAP, mean arterial pressure; LDL-C, low-density lipoprotein cholesterol; HDL-C, high-density lipoprotein cholesterol; TG, triglycerides; HOMA-IR, homeostasis model assessment of insulin resistance.

Subgroup: Overweight group was defined as BMI greater than or equal to 24 kg/m^2^.

### Demographic and Cardiometabolic Risk Factors in Normal Weight and Normal Waist Circumstance Population

All variables in men with MONW and normal WC were significantly different from those in men without MONW and normal WC. All variables, except T-CHOL, in women with MONW and normal WC, were significantly different from those of women without MONW and normal WC. The demographic and cardiometabolic risk factors of participants with normal body weight and normal WC are presented in **Tables 3**, **4**, respectively. Those with a normal BMI (n = 5233) constituted 51.6% of the total population. Of 2327 men with normal BMI (18.5–23.9 kg/m2), 421(18.1%) had insulin resistance, as defined by HOMA-IR ≥ 1.70. Of the 2906 women with normal BMI, 475 (16.3%) had insulin resistance (HOMA-IR ≥ 1.78). Those with normal WC (n = 6832) constituted 67.4% of the total population. Of the 3747 men with normal WC, 983 (26.2%) had insulin resistance, as defined by HOMA-IR ≥ 1.70. Of the women with normal WC (n = 3085), 448 (14.5%) had insulin resistance (HOMA-IR ≥ 1.78).

**Table 3:** Demographic and cardiometabolic risk factors in normal weight (body mass index 18.5 to 23.9 kg/m^2^) adults by HOMA-IR (N=5233) and gender.

Variables	Male			Female		
	HOMA-IR			HOMA-IR		
	<1.70 (n=1906)	≥1.70 (n=421)	p value	<1.78 (n=2431)	≥1.78 (n=475)	p value
Age (year)	38.34 ± 5.10	38.42 ± 5.23	0.79	38.05 ± 5.12	37.91 ± 5.09	0.59
SBP (mmHg)	114.01 ± 13.61	117.83 ± 13.35	<0.001	103.73 ± 11.44	108.21 ± 12.77	<0.001
DBP (mmHg)	71.33 ± 9.52	74.84 ± 9.55	<0.001	63.81 ± 8.54	66.20 ± 9.54	<0.001
Fasting glucose (mmol/L)	4.98 ± 0.71	5.84 ± 2.18	<0.001	4.91 ± 0.42	5.27 ± 0.77	<0.001
Total cholesterol (mmol/L)	5.07 ± 0.91	5.28 ± 0.90	<0.001	4.75 ± 0.80	4.84 ± 0.85	0.04
Triglycerides (mmol/L)	1.32 ± 1.29	1.91 ± 1.30	<0.001	0.80 ± 0.47	1.11 ± 0.65	<0.001
LDL cholesterol (mmol/L)	3.24 ± 0.80	3.41 ± 0.83	<0.001	2.83 ± 0.71	2.98 ± 0.76	<0.001
HDL cholesterol (mmol/L)	1.30 ± 0.29	1.17 ± 0.24	<0.001	1.51 ± 0.31	1.36 ± 0.29	<0.001
TG/HDL-C	1.14 ± 1.67	1.76 ± 1.39	<0.001	0.58 ± 0.54	0.90 ± 0.68	<0.001

BMI, body mass index; SBP, systolic blood pressure; DBP, diastolic blood pressure; MAP, mean arterial pressure; LDL-C, low-density lipoprotein cholesterol; HDL-C, high-density lipoprotein cholesterol; TG, triglycerides; HOMA-IR, homeostasis model assessment of insulin resistance.

**Table 4:** Demographic and cardiometabolic risk factors in normal waist circumstance (men<90cm, women<80cm) adults by HOMA-IR (N=6832) and gender.

Variables	Male			Female		
	HOMA-IR			HOMA-IR		
	<1.70 (n=2764)	≥1.70 (n=983)	p value	<1.78 (n=2637)	≥1.78 (n=448)	p value
Age (year)	38.37 ± 5.13	38.46 ± 4.96	0.64	37.34 ± 5.51	37.85 ± 5.24	0.07
SBP (mmHg)	115.03 ± 14.02	121.18 ± 14.61	<0.001	103.65 ± 11.49	108.64 ± 13.63	<0.001
DBP (mmHg)	72.10 ± 9.87	76.76 ± 10.40	<0.001	63.73 ± 8.64	66.68 ± 9.86	<0.001
Fasting glucose (mmol/L)	4.98 ± 0.67	5.74 ± 1.87	<0.001	4.89 ± 0.42	5.28 ± 0.64	<0.001
Total cholesterol (mmol/L)	5.09 ± 0.91	5.34 ± 0.94	<0.001	4.74 ± 0.83	4.87 ± 0.87	0.004
Triglycerides (mmol/L)	1.39 ± 1.30	2.16 ± 1.74	<0.001	0.78 ± 0.45	1.10 ± 0.65	<0.001
LDL cholesterol (mmol/L)	3.26 ± 0.82	3.44 ± 0.87	<0.001	2.80 ± 0.74	3.00 ± 0.77	<0.001
HDL cholesterol (mmol/L)	1.28 ± 0.29	1.14 ± 0.23	<0.001	1.53 ± 0.32	1.38 ± 0.29	<0.001
TG/HDL-C	1.22 ± 1.68	2.04 ± 1.94	<0.001	0.56 ± 0.51	0.88 ± 0.69	<0.001

BMI, body mass index; SBP, systolic blood pressure; DBP, diastolic blood pressure; MAP, mean arterial pressure; LDL-C, low-density lipoprotein cholesterol; HDL-C, high-density lipoprotein cholesterol; TG, triglycerides; HOMA-IR, homeostasis model assessment of insulin resistance.

## Discussion

In the present study, we observed that the increased risk of MetS in the male and female groups was associated with increased HOMA-IR. We defined the optimal cutoff point of HOMA-IR correlation with MetS as 1.7 and 1.78 for men and women, respectively. Relative to other components, the TG/HDL ratio had a greater and more reliable accuracy for predicting MetS in our study population. Those with normal weight and WC who exhibited IR also had higher BP, fasting glucose, and lipid components in this study.

Previous studies (21, 22) have revealed that IR and MetS are closely associated. Population-based studies (18, 23) have also indicated that the odds of developing MetS increase with increasing HOMA-IR. The number of MetS components in the previous study was directly related to the level of HOMA-IR, and the rising HOMA-IR was related to an increased risk of MetS. This could be explained by insulin resistance being an early clue in the pathogenesis of MetS (8, 23). Nevertheless, cross-sectional studies cannot identify the direction of causality between MetS and HOMA-IR. However, related findings have highlighted that those with a higher HOMA-IR level have a greater risk of being diagnosed with MetS. The optimal cutoff point of HOMA-IR for MetS, as defined by ATP III, in our study was 1.7 for men and 1.78 for women. The sensitivity and specificity of the HOMA-IR cutoffs were 75% and 70% in men and 79% and 81% in women, respectively. A study on the Iranian population (14) revealed that the optimal threshold of HOMA-IR for detecting MetS was 1.85 in men and 1.95 in women. However, the sensitivity (64% in men; 57% in women) and specificity (68% in men and 67% in women) of these HOMA-IR threshold values were not as high as those in our study. In a study in a Thai population aged over 35 years with normal BMI and fasting glucose, the cutoff value of HOMA-IR was 1.55, with the criterion being the 90th percentile or above (24). In a report on healthy Japanese participants, in which the 90th percentile was also used as the criterion, the HOMA-IR cutoff was 1.7 (25). In a report whose HOMA-IR value exceeded the 66th percentile in a US adult population with a normal BMI and fasting glucose, the HOMA-IR cutoff was 2.73 (26). The cutoff value for healthy Italian participants was 2.77, with the 80th percentile as the criterion (27). Diversity in ethnicity and health status has resulted in different HOMA-IR cutoff values for MetS diagnosis (28). The use of different statistical methods or definitions, such as ROC, different percentiles, or quantiles, may also affect the cutoff points for HOMA-IR. Moreover, the lack of a definition for the calculation of IR may affect the cutoff values in populations (14, 28).

In those aged <18 years, HOMA-IR increased according to sexual maturity in both sexes in urban Indian adolescents, and the optimal cutoff value of HOMA-IR was 2.5 for maximal sensitivity and specificity in the total population (19). The optimal value for predicting MetS in Chinese children and adolescents was 2.3 (18). The EPIRCE study (15), a study in a Spanish population without diabetes, reported an optimal value of 2.07 in women aged < 50 years. However, that study also observed a nonlinear effect on the accuracy of HOMA-IR. In Spanish women, sensitivity ranged from 79% in those aged 30 years to 48% in those aged 70 years (15). Differences in body composition, such as adipose tissue distribution, particularly hepatic and visceral adiposity, contributed to differing degrees of IR by sex (29). Because women have greater amounts of peripheral and subcutaneous adipose tissue and benefit from the protective effect of the sex hormone estrogen, they have a more insulin-sensitive body composition. The age effect in women may be related to hormonal changes due to menopause after the age of 50 years (17). The accuracy of HOMA-IR for MetS prediction may not be applicable to older women (15).

To evaluate the predictors of cardiometabolic risk, we depicted areas under the ROC curve, which revealed the sensitivity and specificity for the optimized cutoff points of MetS components. For the prediction of MetS, WC (89.75 cm in men; 79.75 cm in women) and TG/HDL (1.47 in men; 0.86 in women) provided greater accuracy than that by other components in both the male and female groups. This indicates that WC and TG/HDL were the most suitable predictors in this large general population. A study on Brazilian middle-aged men (average age 51 years) concluded that a WC cutoff point of 88 cm was significantly related to MetS, IR, and cardiometabolic risk factors (30). Other studies in Asia have revealed that the optimal cut-off point of WC is in the upper normal range of 85–89 cm and 75–79 cm in men and women, respectively, with normal weight and BMI (31) or 87 cm and 82 cm in the Chinese population with a normal weight. These data were consistent with our findings. MetS is related to obesity; however, it also occurs in the MONW population (11, 24, 32). WC cutoffs established by international organizations, such as the World Health Organization, are used to evaluate cardiometabolic disease risk. Even within populations with normal weight or normal BMI, central obesity has been demonstrated to be a critical predictor for the early diagnosis of MetS. WC is also influenced by ethnicity, age, and sex (31). MetS may occur in Asians with lower WC because they may have a greater percentage of central adipose tissue at a lower weight than their Western counterparts (33, 34). Dissimilar adipose tissue distribution in the male and female body also contributes to their different WC cutoff points (29). Because WC is an accurate predictor of MetS diagnosis, WC is recommended because of its simple measurement and typical inclusion in health visits in hospitals or health checkups in communities. TG/HDL (1.47 in men; 0.86 in women) provided greater accuracy for MetS prediction than other components in both the male and female groups in our study. A study of healthy community-dwelling adults in Japan revealed that in men and women, the TG/HDL ratio was the optimal marker: it was significantly and strongly associated with the MetS variable in multiple linear regression analysis (35). In Americans, the TG/HDL ratio (ROC curve of 0.770) is also a reliable marker for MetS prediction (36).

The notion of MONW suggests that despite having normal body weight, non-obese individuals may still be susceptible to MetS, which includes ailments such as hypertension, dyslipidemia, and coronary artery disease. Impairment of fasting glucose or high IR is a key characteristic of these diseases (15, 32). The possible etiology of MONW is related to excess accumulation of abdominal fat (37). These individuals demonstrated higher central fat mass and lower fat-free mass, which were related to reduced IR in a related study (38). To avoid serious complications related to MetS later in life, early diagnosis and intervention (e.g., lifestyle modification and early pharmacotherapy) in such individuals may be beneficial. A Korean population study revealed that the prevalence of MONW was 14.2% in men and 12% in women aged over 40 years within the highest quartile in the HOMA-IR of 2.05 (39). Another study in Korea revealed that the prevalence of MetS was 10.1% and 7.6% in men and women, respectively, with BMI < 25 kg/m2 (40). In a Polish population study, those with HOMA-IR values of ≥1.69 were classified in the MONW group. The prevalence of MetS in this population was 31.42% in men and 21.76% in women (37). A study in an Iranian population evaluated the lower limit of the top quintile of HOMA-IR values as the optimal cutoff point; the MetS prevalence of those with MONW was 39.8% in women and 33.8% in men with HOMA-IR of ≤2.482 (41).

A wide range of MONW prevalence has been reported (39–41). The lack of a standard criterion for identifying MONW individuals, different cutoff values of HOMA-IR, and other factors such as age, race, and sex contribute to this diversity (37, 41). The biological mechanism of lipid clearance and secretion may contribute to the difference in MetS prevalence between men and women (41). In the present study, T-CHOL was significantly different between men with MONW and normal WC; this difference was not observed in the female group. Sex differences in lipoprotein metabolism have also been reported in other studies (42, 43). The production of triglyceride-rich very low-density lipoproteins (VLDL) and fatty acids was higher in lean women than that in lean men, which contributes to higher lipoprotein lipase activity in women (42, 43). The clearance rate of VLDL-TG in plasma was higher in lean women than in lean men, which may be attributed to the lower VLDL-TG concentration in women (42, 43). In lipoprotein metabolism, insulin plays a role in reducing triglyceride-rich VLDL particles and the availability of fatty acids (44). Women with IR exhibited increased secretion of VLDL and decreased clearance rates of other lipoproteins (41). In a Polish population study (37), higher TG and LDL-C levels but lower HDL-C levels were observed in the group of women with MONW. These findings may corroborate the present results that levels of lipoproteins, such as LDL, TG, and HDL, differ significantly between women with MONW and women with normal weight; however, T-CHOL was not significantly different.

The strength of this study is that a large number of adults without diabetes in a Chinese population were analyzed. We were able to determine a precise cutoff value of HOMA-IR for young Chinese adults, a group that has received comparatively little research attention. We also used HOMA-IR, a simple measurement of IR, and it exhibited a favorable correlation with insulin sensitivity in our study. This finding may be applied in the early diagnosis of cardiometabolic risk in clinical settings. However, our study had some limitations. The cross-sectional nature of this study means that we cannot draw conclusions regarding causality between cardiometabolic risk factors and IR. Other variables, such as lifestyle habits, were not analyzed: these variables may be a source of bias or limiting factors. More prospective studies are required to clarify this matter further.

## Conclusions

We established the optimal cutoff point of HOMA-IR correlation with MetS; that is, 1.7 and 1.78 in the male and female population, respectively, in this study. Those with normal weight and WC in our population who also had IR exhibited higher BP, fasting glucose, and lipid components. The findings of this study would provide an evidence-based threshold for evaluating metabolic syndromes and further implementing primary prevention programs, such as lifestyle changes in the target population.

## Data Availability Statement

The original contributions presented in the study are included in the article material, further inquiries can be directed to the corresponding author.

## Ethics Statement

The study data from the health check-up records of Chinese adults between 2013 and 2016. The study design was approved by the appropriate ethics review board of Xiamen Chang Gung hospital.

## Author Contributions

W-CL and J-YC help in conception,design of the study and in commenting on a draft of the chapter. The quality of this experiment was greatly enhanced by the gracious assistance of them. S-YL and W-CL contributed equally to this work. T-AY, Y-CC, and X-JX prepared the material and data collections. H-YH and WY performed the analysis. Szu-Yu Lin wrote the first draft of the manuscript. W-CL and J-YC revised the manuscript. All authors contributed to the article and approved the submitted version.

## Conflict of Interest

The authors declare that the research was conducted in the absence of any commercial or financial relationships that could be construed as a potential conflict of interest.

## Publisher’s Note

All claims expressed in this article are solely those of the authors and do not necessarily represent those of their affiliated organizations, or those of the publisher, the editors and the reviewers. Any product that may be evaluated in this article, or claim that may be made by its manufacturer, is not guaranteed or endorsed by the publisher.
